# Perpendicular Structure Formation of Block Copolymer Thin Films during Thermal Solvent Vapor Annealing: Solvent and Thickness Effects

**DOI:** 10.3390/polym9100525

**Published:** 2017-10-18

**Authors:** Qiuyan Yang, Katja Loos

**Affiliations:** Macromolecular Chemistry & New Polymeric Materials, Zernike Institute for Advanced Materials, University of Groningen, Nijenborgh 4, 9747 AG Groningen, The Netherlands; yangqiuxiaoyan@163.com

**Keywords:** block copolymers, thin films, annealing, selectivity, lamellar

## Abstract

Solvent vapor annealing of block copolymer (BCP) thin films can produce a range of interesting morphologies, especially when the perpendicular orientation of micro-domains with respect to the substrate plays a role. This, for instance, allows BCP thin films to serve as useful templates for nanolithography and hybrid materials preparation. However, precise control of the arising morphologies is essential, but in most cases difficult to achieve. In this work, we investigated the solvent and thickness effects on the morphology of poly(styrene-*b*-2 vinyl pyridine) (PS-*b*-P2VP) thin films with a film thickness range from 0.4 *L*_0_ up to 0.8 *L*_0_. Ordered perpendicular structures were achieved. One of the main merits of our work is that the phase behavior of the ultra-high molecular weight BCP thin films, which hold a 100-nm sized domain distance, can be easily monitored via current available techniques, such as scanning electron microscope (SEM), atomic force microscope (AFM), and transmission electron microscope (TEM). Systematic monitoring of the self-assembly behavior during solvent vapor annealing can thus provide an experimental guideline for the optimization of processing conditions of related BCP films systems.

## 1. Introduction

The self-assembly of block copolymer (BCP) thin films can generate structures with a tailored length, enabling them to be desirable templates for nano-manufacturing [1]. Controlling the morphology of ordered BCPs was normally achieved via thermal annealing or solvent vapor annealing (SVA) [2,3]. In SVA, as-prepared block polymer thin films are exposed to vapors of one or more solvents at temperatures typically well below the bulk *T*_g_ of both blocks to form a swollen and mobile polymer film on the substrate [4]. This process is highly effective over comparatively short timescales, particularly for high molecular weight BCPs in comparison to thermal annealing, due to the dramatic enhancements of chain mobility afforded by the presence of a plasticizing solvent in the BCP film as compared to the thermal-activation of chain motion in a dry melt [1]. Such rapid assembly kinetics in SVA and the versatility of the technique motivate great efforts in this area. Among reported BCPs thin films, the self-assembly of poly(styrene-*b*-vinyl pyridine) (PS-*b*-PVP) films under SVA was particularly studied because of the intrinsically high Flory–Huggins segmental interaction parameter (*χ*) for phase separation and the functionality of the vinylpyridine group with small molecules and nanoparticles for the further fabrication of hybrid materials. For example, various arrays including micellar, laminar, and cylindrical domains of PS-*b*-P4VP with a high degree of orders were produced by Park and Russell, which were further used as the templates for the fabrication of porous films [5], dot and stripe arrays [6,7], metallic line patterns [8], and ordered structure designs [9,10,11].

However, SVA is a more complex process than thermal annealing. The morphology in this process not only depends on the intrinsic properties of BCP, such as the extent of *χ*, the degree of polymerization (*N*), and the composition (block ratio *f*), but also on type of the solvent [12,13,14,15,16,17,18,19] and the film thickness [20,21,22]. Generally, preferentially wetting of one block at the substrate surface or free surface on the contrary induces preferential parallel orientation of domains [22]. For example, Hasegawa et al. showed that the use of nonselective tetrahydrofuran (THF), selective solvent acetone for P2VP domain, or selective solvent toluene for the PS domain in the annealing of thin films PS-*b*-P2VP could cause the BCP to form significantly different morphologies, further asserting that various perpendicular structures can be formed predominantly on the substrates with the proper choice of solvent [23,24]. In addition to the interfacial interaction greatly influenced by the solvent vapor in SVA systems, the thickness of BCP films also affects the orientation of domains [20,21,22,25]. Chang et al. investigated the phase behaviors of polystyrene-block-polyisoprene (PS-*b*-PI) thin films and constructed a phase diagram as a function of film thickness over the range of 150−2410 nm (7−107 *L*_0_ (*L*_0_: domain spacing)) and temperature. In the presence of solvent vapor, commensurability between the swelled film thickness and *L*_0_ is found to be essential to produce a well-ordered self-assembled morphology [26].

Despite the great progress made on the control of the morphology of BCP during the past decades and the more comprehensive understanding of SVA process that is established, a small change on one parameter normally requires corresponding adjustments on other conditions to maintain the morphology, because self-assembly through SVA process is actually a systematic work based on many multivariate factors. Current controlling data in this area are already abundant but still far from sufficient to build up an integrated handbook to guide the structural formation of BCP thin films. Systematic investigations on the phase behavior of BCP are still required in some cases, for example, BCPs with high molecular weight are not yet well reported. Even though BCPs with an ultra-small period have received the most attention for nanolithography, self-assembled structures from high molecular weight BCPs hold feature sizes above a hundred on the nanometer scale and are also in demand for applications such as photonic crystals, solar cells, and desalination membranes [26]. 

Here, the self-assembly behaviors of high molecular weight poly(styrene-*b*-2 vinyl pyridine) (PS-*b*-P2VP) BCP with *M*_n_(PS) = 133 kg mol^−1^, *M*_n_(P2VP) = 132 kg mol^−1^ (*f*_ps_ ≈ 0.5), and *M*_w_*/M*_n_ = 1.15 during the SVA process are monitored in a detail. A thickness range of 0.4 *L*_0_ to 0.8 *L*_0_ (i.e., from 60 nm to 100 nm) avoiding extra layer formation on the top of thin films was specifically studied here to achieve the perpendicular structures of PS-*b*-P2VP. With the aim of fabricating ordered and reproducible BCP structures in a controllable way, a series of systematic studies on the SVA produced morphologies as functions of solvent type, temperature, film thickness, and annealing time were investigated.

## 2. Materials and Methods

### 2.1. Materials

PS-*b*-P2VP was purchased from Polymer Source, Inc. and used as received: P5742-S2VP, *M*_n_ (PS) = 133 kg mol^−1^, *M*_n_ (P2VP) = 132 kg mol^−1^, *M*_w_*/M*_n_ = 1.15. Toluene Biosolve Chemicals (Biosolve BV, Valkenswaard, The Netherlands), >99.7%) and chloroform (Biosolve Chemicals, >99.9%) were used as received Silicon substrates (Prime CZ-Si wafer, thickness = 625 ± 20 μm, (100), 1-sidepolished, p-type (Boron) TTV < 10 μm, 15–25 Ohm) were supplied by Micro Chemicals GmbH, Ulm, Germany. 

### 2.2. Preparation of PS-b-P2VP Thin Films

PS-*b*-P2VP thin films were prepared by spin-coating of the polymer solution onto 1 cm × 1 cm silicon wafer. Silicon (Si) substrates of 1 cm^2^ with a native silicon oxide layer on the surface were ultrasonically cleaned in water, ethanol, and acetone for 10 min (three times for each type of solvent), respectively, and dried under a stream of air gas before use. The concentration of the polymer in solution and also the spin-coating speed were adjusted with a couple of trial experiments to yield films with the desired thickness and different morphologies, as shown in Table 1. 

The obtained films were dried under vacuum to remove the retained solvent before further solvent vapor annealing, especially when different solvent vapors were applied for annealing. However, the as-spin-coated sample was directly used for solvent vapor annealing if the solvent vapor was the same as that of the casting solvent. SVA treatments were conducted in a 60-mL glass container filled with solvent vapors by placing the spin-coated films at the bottom. One open small 4-mL bottle, which was typically filled with 2 mL of solvent, was placed inside this container to yield the solvent vapor. By closing the lid of the container and putting it inside a chamber with proper temperature control, the relative solvent vapor pressure inside the dish could roughly be adjusted, resulting in the swelling of the films to different swelling ratios. Solvent vapor annealing was conducted in chloroform at 27 °C and in toluene at temperatures from 50 to 80 °C with various annealing times with the aim of obtaining ordered morphologies of block copolymers.

Thin films of PS-*b*-P2VP prepared by spin-coating were floated off of the silicon substrates by immersing the samples in a 1 wt % NaOH aqueous solution for half an hour, after which they were dried on copper grids for TEM imaging.

### 2.3. Characterization

Atomic Force Microscopy (AFM) measurements were carried out on a Digital Instruments Enviro Scope AFM equipped with a Nanoscope III controller in tapping mode using Veeco RTESPW silicon cantilevers (*f*_0_ = 240–296 kHz and *k *= 20–80 N m^−1^ as specified by the manufacturer). AFM images were typically obtained with a scan range of 10 and 3 μm^2^ and a frequency of 0.5 Hz/line. Film thicknesses were measured by scratching the film in three different places with a razor blade followed by scanning across the scratch edges. In this case, the aspect ratio was 1:8 to minimize distortion along the slow scanning axis due to thermal drift. Transmission electron microscopy (TEM) was carried out on a Philips CM12 transmission electron microscope (Philips, Amsterdam, The Netherlands) operating at an accelerating voltage of 120 kV. TEM images were recorded on a Gatan slow-scan charge-coupled-device (CCD) camera.

## 3. Results and Discussion

**Figure 1:**
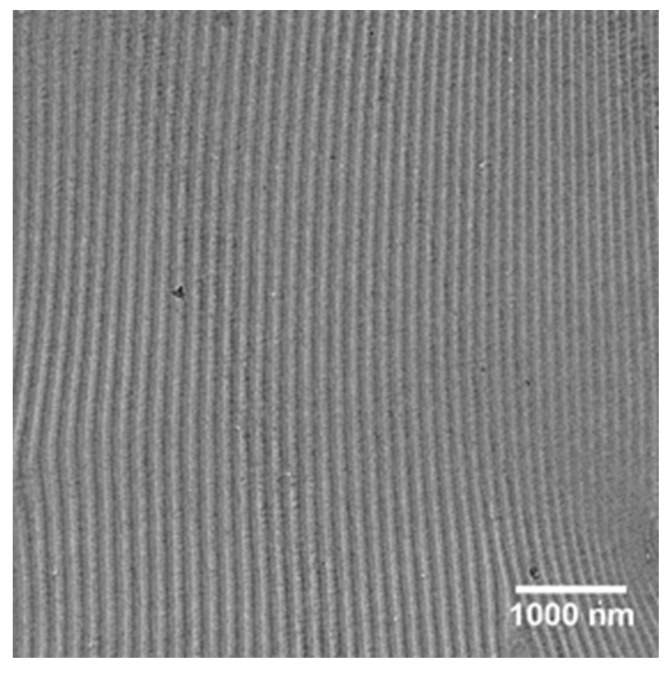
TEM image of the PS-*b*-P2VP sample with a thickness over 45 *L*_0_ annealed in chloroform vapor atmosphere at 27 °C for a week.

A PS-*b*-P2VP BCP with a molecular weight *M*_n_(PS) = 133,000 g mol^−1^, M_n_(P2VP) = 132,000 g mol^−1^ ( *f*_ps_ ≈ 0.5), and *M*_w_*/M*_n_ = 1.15 is chosen. Contributed by its high *χN* (≥100) value, a lamellar spacing (*L*_0_) around 146 nm is observed in the TEM image of an annealed BCP sample with a thickness higher than 45 *L*_0_ (Figure 1). The PS-*b*-P2VP copolymer exists as spherical micelles (with a PS corona and a P2VP core) in toluene solutions due to the favorable interaction with the PS domains [27,28,29,30] and is normally shown as milk white in color in the solutions. On the contrary, in the presence of chloroform, PS-*b*-P2VP solution is absolutely transparent as observed by eye, even at a high concentration of 2.0 wt %, suggesting the better solubility of PS-*b*-P2VP in chloroform than in toluene, which can be easily explained by the Hildebrand solubility parameters, as shown in Table 2.

One of the advantages of the spin-coating technique for thin film preparation is that the thickness (*d*) is controllable, depending on the spin-coating conditions applied, as listed in Table 1 of the experimental section. Silicon (Si) wafers with a native silicon oxide layer on the surface, which are normally considered as preferentially wet P2VP blocks, [32] were used substrates for the spin-coating of the thin film, followed by the SVA processes of as-prepared thin films conducted by enclosing the sample in a temperature-controlled, closed container that contains a small reservoir of liquid solvent. However, the perpendicular orientation, particularly for relatively thick BCP films, suffers from the parallel orientation if there is preferential segregation of one block at the free surface in respect to the other block [25]. A thickness range of 0.4 *L*_0_ to 0.8 *L*_0_ (i.e., from 60 to 100 nm) was specifically studied here in order to avoid multilayer formation on the top of the substrate, in which case phase behaviors can also be easily monitoring monitored by Atomic Force Microscopy (AFM) and Transmission electron microscopy (TEM) during the SVA process.

However, the final morphologies of BCP thin films are actually the results of a balance between interfacial interactions (with the substrate and free surface) of block components during the SVA processes. When different types of solvents were employed, surface interactions of PS-*b*-P2VP blocks with solvent vapor can be varied and thus are expected to lead to various ordered architectures of BCP formed [22]. Therefore, the effects of two different types of solvents, i.e., the selective solvent toluene and the less-selective solvent chloroform (Table 2), were particularly studied.

**Figure 2:**
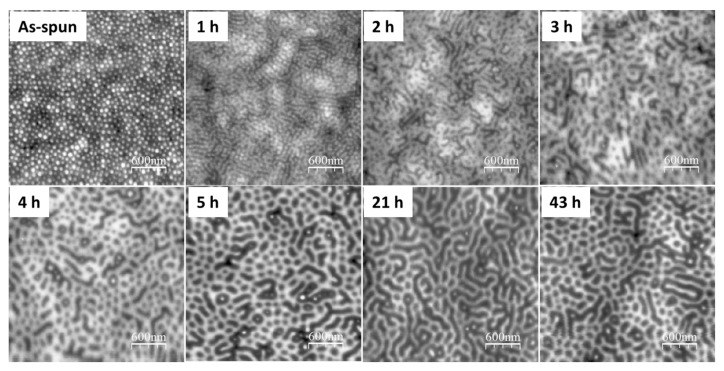
AFM surface height profiles of 60 nm PS-*b*-P2VP thin films spin-coated at 3000 rpm from 1.5 wt % solution in toluene with further annealing under toluene vapor atmosphere at 50 °C with different annealing times.

**Figure 3:**
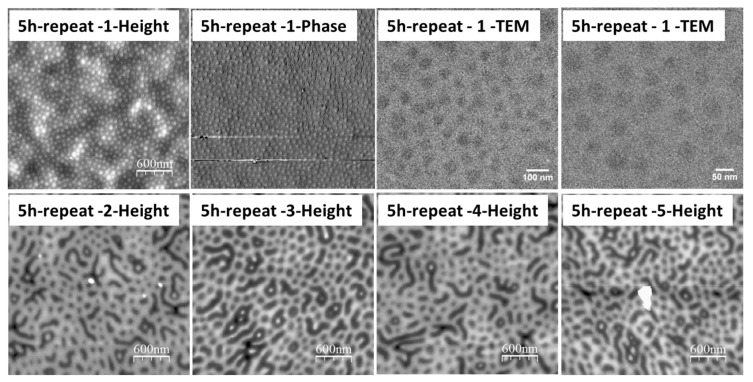
AFM surface profiles and TEM images of the 60-nm PS-*b*-P2VP thin films spin-coated at 3000 rpm from 1.5 wt % solution in toluene with further annealing under toluene vapor atmosphere at 50 °C for 5 h with several repeated experiments.

**Figure 4:**
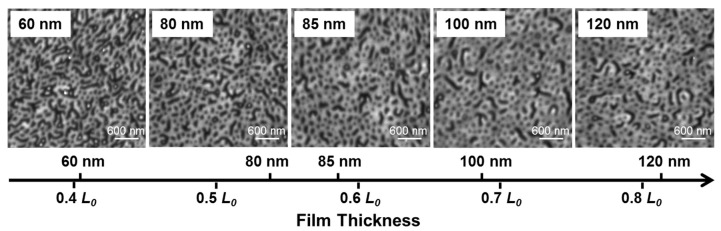
AFM surface height profiles of PS-*b*-P2VP thin films with different thicknesses annealed under toluene vapor atmosphere at 50 °C for 3 h.

**Figure 5:**
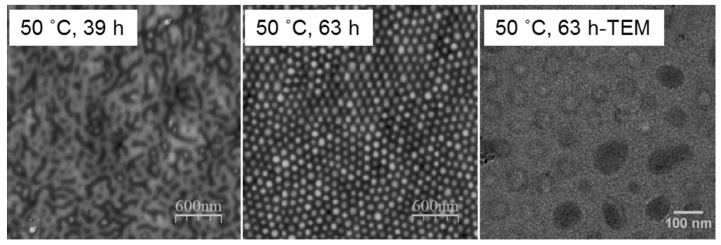
AFM height profiles and TEM images of the 80-nm PS-*b*-P2VP thin films spin-coated at 2500 rpm from 1.5 wt % solution in toluene with further annealing in toluene vapor atmosphere at 60 °C with different annealing times.

In the presence of the selective solvent vapor, in this case toluene, the PS block of the PS-*b*-P2VP can be preferentially swollen, which results in a reconstruction of the films. The solvent pressure in a closed system is expected to reach an equilibrium value in a short period of time, and thus prolonging the annealing time can provide sufficient time for PS chain movement at a given temperature. As shown in the AFM height profiles of the 60-nm (thickness *d *≈ 0.4 *L*_0_) thin film from Figure 2, initial spherical micellar structures were changed into various morphologies with the increase of the annealing time at 50 °C. However, only locally ordered structures were formed in the time length scale up to 43 h because the movements of block copolymers for such thin films were greatly trapped by the interaction between the P2VP block and the oxidized silicon substrate [21,33,34,35]. Even though cylindrical morphologies perpendicular to the substrate was trapped once during repeated experiments at the annealing time for 5 h, the observed hexagonal ordered array encountered problems when it was attempted to be repeated using the same annealing procedure (Figure 3), probably because the resulting non-equilibrium morphology from the SVA process is a metastable state and sensitive to a range of other parameters including the swelling ratio, as well as swelling and de-swelling rates. No obvious difference in the surface morphology was observed when increasing on the thickness of PS-*b*-P2VP films from 60 to 120 nm (0.4 *L*_0_ to roughly around 0.8 *L*_0_) (Figure 4) when annealing thin films for 3 h at 50 °C. With further prolonging the annealing time to 63 h, however, the thicker 80-nm film (0.5 *L*_0_) formed more ordered micelle-like structures (Figure 5), suggesting the possibility of achieving even more ordered structures via adjusting the annealing conditions, such as increasing the annealing time or the vapor temperature.

**Figure 6:**
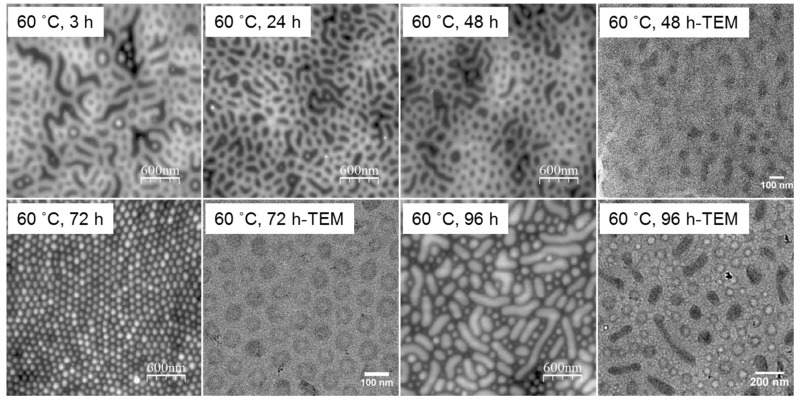
AFM height profiles and TEM images of the 80-nm PS-*b*-P2VP thin films spin-coated at 2500 rpm from 1.5 wt % solution in toluene with further annealing in toluene vapor atmosphere at 60 °C.

**Figure 7:**
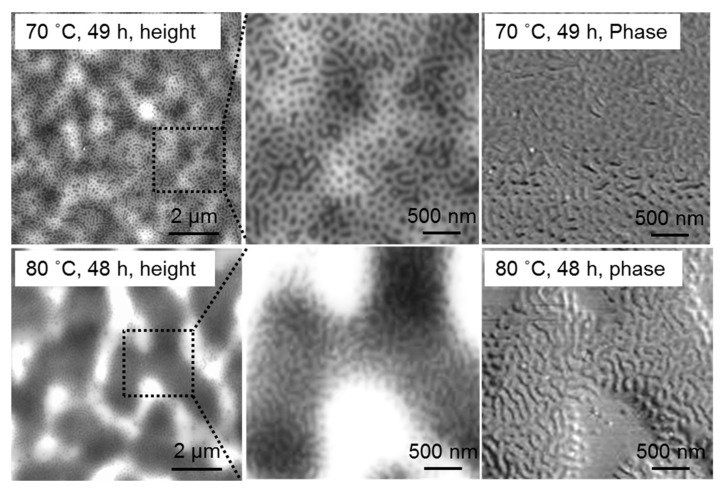
AFM profiles and TEM images of the 80-nm PS-*b*-P2VP thin films spin-coated at 2500 rpm from 1.5 wt % solution in toluene with further annealing in toluene vapor atmosphere at 70 °C and 80 °C.

High temperature can give rise to better diffusivity of the highly entangled polymer chains, which would greatly decrease the annealing time length scale. As displayed in surface AFM images and cross-sectional TEM images of obtained thin film structures in Figure 6, obvious phase transition of PS-*b*-P2VP films with a pathway of micelles-perpendicular cylinders (~45 nm)—micelles-parallel cylinders was demonstrated. Specifically, highly ordered micelle-like structures with the micelle size around 100 nm were formed at 60 °C, 72 h among these annealing processes. The state became less ordered at annealing times above 72 h, such as at 96 h. In principle, both vapor pressure and thermal chain mobility can be increased by increasing the temperature from 60 °C to a higher temperature, and thus ordered structures can be expected in a shorter time. However, further increasing the annealing temperature did not improve the ordering degree of films, as normally occurs [36]. On the contrary, thin films were separated into thinner and thicker regions at higher annealing temperatures like 70 or 80 °C (Figure 7), an effect known as terraces formation [37,38,39,40], due to the obvious change in the surface energy and thus the overall morphology at high temperature region. Meanwhile, the microstructure at the slope between the islands of different thicknesses was still lamellar at the temperature of 80 °C. 

**Figure 8:**
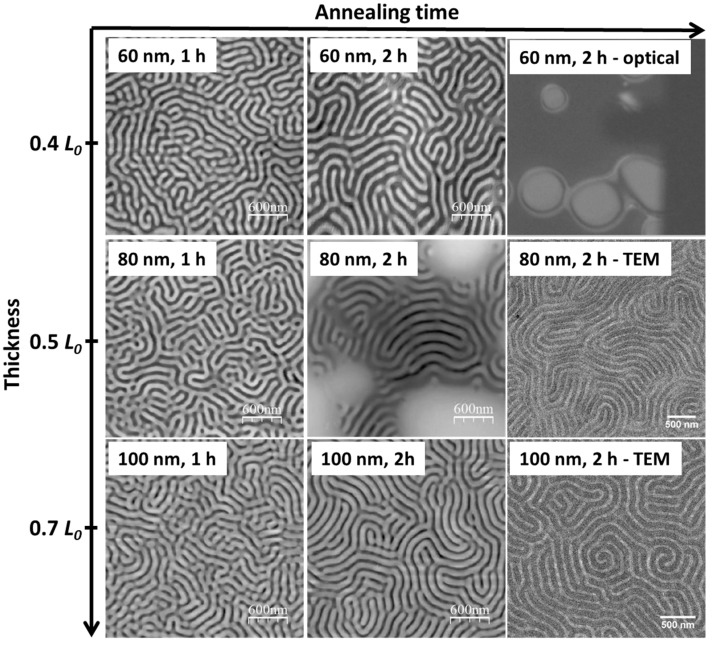
AFM surface height profiles, optical microscope image (upper-right corner), and TEM images of the PS-*b*-P2VP thin films annealed in chloroform vapor atmosphere at 27 °C for different time periods.

Accordingly, to further increase the order degree of perpendicular structures, the interaction of PS-*b*-P2VP blocks with free surfaces have to be greatly increased to thus neutralize the substrate interaction. Chloroform, a less selective solvent for PS-*b*-P2VP along with higher vapor pressure as compared to toluene (Table 2), is thus further used as the vapor solvent, in which case much higher chain mobility can also be expected. The AFM images in Figure 8 demonstrate that ordered structures with fingerprint-like lamellar surface morphologies were formed in quite short annealing periods for all films with thicknesses from 60 to 100 nm. Similar to the toluene system, the longer the annealing time applied, the higher the degree of phase separation achieved. Generally, a thinner film takes a shorter time to form ordered structures. The cross-sectional TEM images of 80- and 100-nm thick PS-*b*-P2VP films confirmed the one-layer structures perpendicular to the substrate, indicating a balance between the free surface and substrate interaction for PS-*b*-P2VP in the presence of chloroform at the given conditions. For the same reasons, P2VP chains from the substrate interface and PS segments from the free surface move into the BCP phases, respectively, contributing to the increase of the domain sizes for thicker films at the same annealing conditions.

**Figure 9:**
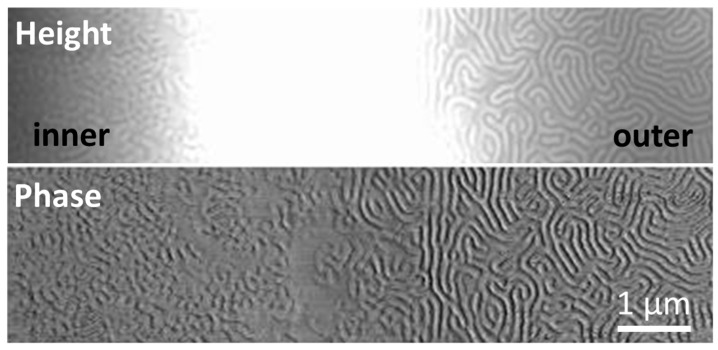
AFM surface height and phase profile (aspect ratio was 1:8) for the edge region of the defect ring from the 60-nm PS-*b*-P2VP thin films annealed under chloroform vapor atmosphere at 27 °C for 120 min.

It should be mentioned that the thickness of the film must be carefully considered because much thinner films can be easy to dewet, especially if the films were exposed to a solvent atmosphere with high vapor pressure, such as a chloroform atmosphere. For example, ring-like micron-sized defects (white circular regions in the upper-right image of Figure 8) were formed for 60-nm thick films (*d *≈ 0.4 *L*_0_) when they were annealed just for 2 h. Even though the microstructures are fingerprint-like lamellar in the major dark region, structures other than perpendicular lamellar structures were found (Figure 9) within the defect and also in the slope of these defects. Also, extra layers were observed for 80-nm films (*d *≈ 0.5 *L*_0_) (Figure 9) annealed for 2 h. The formation of terraces (i.e., islands or holes) in these two samples actually depends on the interaction of the blocks with the substrate and the vapor [21]. In particular, when the thickness of a film is incompatible with this inherent length scale, non-favorable interactions of the solvent with the substrate could result in terraces. The phenomena can be avoided by further increasing the film thickness to 100 nm, suggesting a synergistic effect between the film thickness and the annealing time. 

## 4. Conclusions

Phase behaviors of PS-*b*-P2VP thin films with thicknesses ranging from 0.4 *L*_0_ up to 0.8 *L*_0_ were investigated. In these cases, perpendicular structures in regard to the substrate were normally formed, which were found to be very sensitive to the solvent vapors. A strong selective solvent, such as toluene for PS-*b*-P2VP, can preferentially swell one block (PS) and thus change the effective block “composition” and possibly also the “equilibrium” state of the BCP (forming cylinders and micelles rather than a lamellar morphology) during the slow phase transition process. In contrast to this, a less selective solvent vapor, such as chloroform vapor for PS-*b*-P2VP, can balance the interactions of the free surface and substrate of these two blocks, and thus result in morphologies close to the bulk equilibrium state (fingerprint-like lamellar morphologies in our case) in quite short annealing periods. Terraces were formed in thinner films as compared to thicker ones at given time length scales, particularly in a thickness range below the inherent length scale of the BCP (i.e., *d *< *L*_0_). One of the main merits of our work is that the phase behavior of the ultra-high molecular weight BCP thin films, which hold above 100-nm sized domain distances, can be easily monitored via current available techniques, such as SEM, AFM, and TEM, which are expected to provide experimental guidelines for the optimization of treatment conditions during the SVA processes of other BCPs. 

## Figures and Tables

**Table 1:** Spin-coating conditions for various PS-*b*-P2VP films with different thicknesses.

Solvent	Concentration (*w/v* %)	Spin-Coating Speed (rpm)	Film Thickness (nm)
Toluene	1.5	3000	~60
	1.5	2500	~80
	1.5	2000	~85
	2.0	3000	~100
	2.0	2000	~120

**Table 2:** Vapor pressure (VP), Hildebrand solubility parameter (δ), and enthalpy derived interaction parameter ($\chi_{H}^{\infty }$), relative to the individual blocks and the copolymer, for toluene and chloroform at 273.15 K.

Solubility Parameters...	Toluene	Chloroform	PS	P2VP
Vapor Pressure (mm/Hg)	21.9	156.05	-	-
Molar voume (cm^3^/mol)	105.7	79.5	-	-
δ ((cal/cm^3^)1/2) ^a^	18.3	18.7	18.5	20.6
$\chi_{H}^{\infty }$ (PS) ^b^	0.00	0.00	-	-
$\chi_{H}^{\infty }$ (P2VP)	0.11	0.08	-	-

^a^ Hildebrand solubility parameter (δ) [31]; ^b^ Solvent–polymer interactions can be predicted from the solvent parameter on the basis of complementary matching and an interaction parameter (enthalpy related and at infinite dilution), $\chi_{H}^{\infty }$ given by(Vi/RT)(δ_i_−δ_j_)^2^ where V is the molar volume and i and j (δ_i_ and δ_j_) represent the solubility parameters of the two components [31].
